# New Cerebral Lesions at Magnetic Resonance Imaging after Carotid Artery Stenting Versus Endarterectomy: An Updated Meta-Analysis

**DOI:** 10.1371/journal.pone.0129209

**Published:** 2015-05-27

**Authors:** Giuseppe Gargiulo, Anna Sannino, Eugenio Stabile, Cinzia Perrino, Bruno Trimarco, Giovanni Esposito

**Affiliations:** Division of Cardiology—Department of Advanced Biomedical Sciences, Federico II University, Naples, Italy; Ferrarotto Hospital, University of Catania, ITALY

## Abstract

**Background:**

Carotid endarterectomy (CEA) or stenting (CAS) are associated with a relatively low rate of clinical events, but diffusion-weighted imaging (DWI) is increasingly being used to compare the incidence of new ischemic lesions. Therefore, we conducted an updated meta-analysis on the occurrence of post-procedural new DWI lesions after CAS versus CEA.

**Methods and Results:**

MEDLINE, Cochrane, ISI Web of Science and SCOPUS databases were searched and 20 studies (2 randomized and 18 non-randomized) with a total of 2104 procedures (CAS = 989; CEA = 1115) were included. The incidence of new DWI cerebral lesions was significantly greater after CAS than CEA (40.3% vs 12.2%; 20 studies; 2104 patients; odds ratio [OR] 5.17; 95% confidence interval [CI], 3.31-8.06; p<0.00001). Also peri-procedural stroke (17 studies; 1833 patients; OR 2.01; 95% CI, 1.14-3.55; p=0.02) and stroke or TIA (17 studies; 1833 patients; OR 2.40; 95% CI, 1.42-4.08; p=0.001) were significantly increased after CAS. This latter clinical advantage in the CEA group over CAS was tempered when CEA procedures were performed with shunting in all instead of selective shunting or when CAS was performed with only closed cell stents instead of both closed and open cell stents, however, no significant differences between subgroups emerged.

**Conclusions:**

CAS is associated with an increased incidence of post-procedural brain DWI lesions. This greater amount of ischemic burden may also reflect a higher rate of cerebral events after CAS. However, whether recent technical advances mainly for CAS could potentially reduce these ischemic events still remains to be evaluated.

## Introduction

Carotid artery revascularization in patients with carotid artery disease has the aim of preventing stroke. Carotid endarterectomy (CEA) is the standard treatment for severe asymptomatic and symptomatic carotid stenoses, but in the last years carotid artery stenting (CAS) has increasingly emerged as minimally invasive alternative to surgery [1]. Although in recent studies CAS has shown non-inferiority to CEA in the prevention of stroke, its role remains still highly debated [2–4]. One of the main issues related to CAS seems to be the occurrence of peri-procedural brain ischemic events. Given the relatively small number of clinical events after CEA and CAS, large cohorts of patients are needed for a reliable comparison of the 2 procedures. Therefore, diffusion-weighted magnetic resonance imaging (DW-MRI) has been shown to be a sensitive tool in identifying new ischemic cerebral lesions and has been extensively used in the last years as surrogate marker of stroke in the evaluation of patients undergoing to CEA or CAS [5]. Moreover, major promising technology advancements in CAS techniques have been achieved in the last few years and DWI has been used to compare effectiveness of new implementations to CAS procedures [6, 7].

The aim of this meta-analysis is to provide updated evidence on the incidence of new brain lesions after CAS compared with CEA as detected by DWI.

## Methods

### Study selection

**Fig 1:**
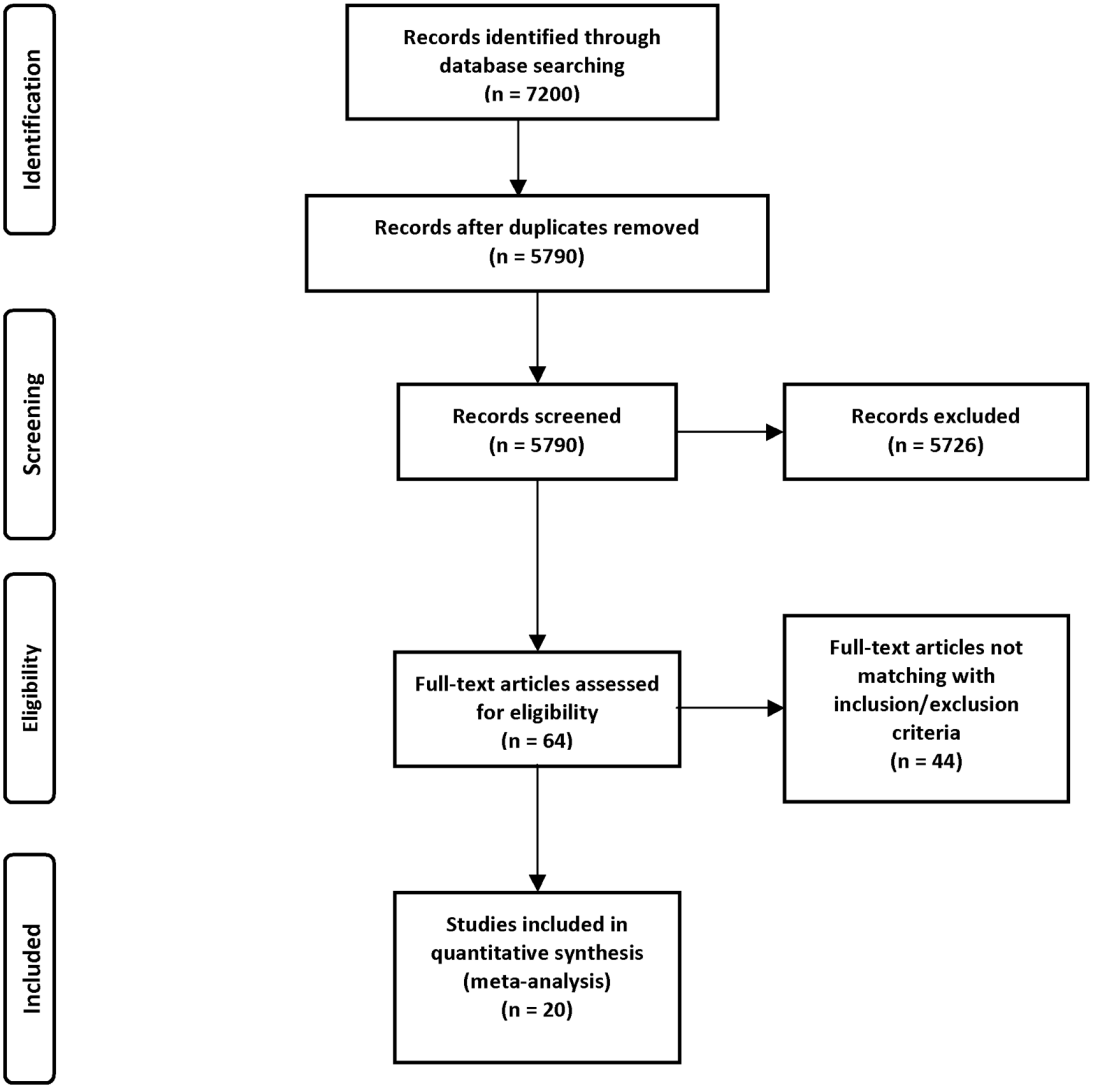
Flow diagram. Studies included in the meta-analysis.

The study was designed according to PRISMA (Preferred Reporting Items for Systematic Reviews and Meta-Analyses) requirements (Fig 1, Table A in S1 file). Articles published until 1^st^ March 2015 were searched in MEDLINE, Cochrane, ISI Web of Science and SCOPUS databases. A combination of the following keywords was used: “carotid stenosis”, “carotid endarterectomy”, “CEA”, “carotid artery stenting”, “CAS”, “carotid angioplasty”, “ischemic lesion”, “cerebral embolism”, “diffusion-weighted imaging”, “DWI”, “magnetic resonance imaging” and “MRI”. Two independent reviewers screened citations at the title and abstract level, and as a full report if reporting data of interest. No language limitations were applied. The full texts and bibliography of all potential articles were also retrieved in detail to search for additional relevant studies.

### Eligibility criteria

Studies were included if both criteria were respected: 1) presence of comparison between CAS versus CEA; 2) presence of post-operative incidence of cerebral lesions detected by DWI-MRI in both groups. Exclusion criteria were: 1) duplicate publication; 2) the outcome of interest was not clearly reported or could not be derived from the published results.

### Data extraction

Two reviewers independently screened the articles for eligibility. The reviewers compared the selected studies and any discrepancy was resolved by consensus. The number of events of new brain lesions at DWI-MRI, stroke and stroke or transient ischemic attack (TIA), number of participants and the main clinical and procedural characteristics were extracted from each study.

### Study endpoints

The primary endpoint was the incidence of new brain lesions after CEA or CAS. Secondary endpoints of interest were stroke and stroke or TIA.

### Statistical analysis

The number of events and participants were abstracted. Estimates of effect were calculated with random-effects model and confirmed by a fixed-effects model and expressed as odds ratio (OR) and risk difference (RD). Statistical significance was set at p < 0.05 (2-tailed). Heterogeneity was assessed by a Q-statistic and I^2^ test. Significant heterogeneity was considered present for p values< 0.10 or an I^2^> 50%. Meta-regressions were performed to test the influence of baseline characteristics (age, male sex, hypertension, diabetes, dyslipidemia, smoke, coronary artery disease, symptoms and year of publication) as potential effect modifiers (significance at p < 0.05). Publication bias was assessed using funnel plots, Egger’s test and trim and fill method. All data analyses were performed using Reviewer Manager (RevMan) Version 5.2 and Prometa Software Version 2 [6–13].

## Results

Of 7200 articles identified by the initial search, 64 were retrieved for more detailed evaluation. Twenty studies directly comparing CAE and CEA and providing the number of new brain lesions at DWI were finally included in the analysis (Fig 1) with a total of 2104 procedures (CAS = 989; CEA = 1115). Two studies were randomized [14, 15], while 18 were non-randomized [16–33]. Characteristics of the studies included are detailed in Table 1.

**Table 1:** Characteristics of the studies included in the meta-analysis.

	**Year**	N*	CAS	CEA	RCT	Age	Men	Hypertension	Diabetes	Dyslipidemia	Smoke	CAD	Symptoms	EPD	Stent type	CEA procedure
Flach et al^[^ ^18^ ^]^	2004	44	21	23	No	69	80%	-	-	-	-	-	100%	Yes	Both	Selective shunting
Garcia-Sanchez et al^[^ ^19^ ^]^	2004	20	10	10	No	66	95%	70%	40%	65%	80%	15%	100%	No	Closed cell	Shunting in all
Poppert et al^[^ ^23^ ^]^	2004	129	41	88	No	69	68%	87%	33%	58%	38%	35%	47%	No	Closed cell	Shunting in all
Roh et al^[^ ^25^ ^]^	2005	48	22	26	No	63	96%	-	-	-	-	-	77%	No	Closed cell	Shunting in all
Lihara et al^[^ ^20^ ^]^	2006	231	92	139	No	69	91%	74%	37%	14%	48%	35%	54%	Yes (all)	Both	Selective shunting
Faraglia et al^[^ ^16^ ^]^	2007	75	35	40	No	70	72%	83%	23%	33%	78%	27%	25%	Yes (all)	Both	Selective shunting
Lacroix et al^[^ ^21^ ^]^	2007	121	61	60	No	72	-	-	-	-	-	-	51%	Yes (all)	Closed cell	Shunting in all
Tedesco et al^[^ ^27^ ^]^	2007	47	27	20	No	68	100%	85%	40%	81%	78%	60%	62%	Yes (all)	Open cell	Selective shunting
Posacioglu et al^[^ ^24^ ^]^	2008	115	56	59	No	66	71%	88%	26%	36%	52%	52%	53%	Yes (all)	Closed cell	No shunting
Skjlland et al^[^ ^26^ ^]^ **	2009	58	28	30	No	66	81%	-	-	-	43%	-	53%	Yes (all)	-	Shunting in all
Zhou et al^[^ ^30^ ^]^	2009	168	68	100	No	-	-	-	-	-	-	-	-	Yes (all)	Both	Shunting in all
Bonati (ICSS-MRI) et al^[^ ^11^ ^]^	2010	231	124	107	Yes	60	70%	69%	21%	64%	76%	22%	100%	Yes (45%)	-	-
Capoccia et al^[^ ^14^ ^]^	2010	43	23	20	No	71	63%	86%	49%	43%	52%	36%	0%	Yes (all)	Closed cell	Selective shunting
Mitsuoka et al^[^ ^22^ ^]^	2011	45	20	25	No	-	-	-	-	-	-	-	87%	Yes (all)	Both	-
Wasser et al^[^ ^28^ ^]^	2011	49	21	28	No	67	79%	88%	37%	77%	58%	20%	53%	Yes (38%)	-	Selective shunting
Yamada et al^[^ ^29^ ^]^	2011	81	56	25	No	72	89%	82%	40%	54%	-	39%	62%	Yes (all)	Both	Shunting in all
Akutsu et al^[^ ^13^ ^]^	2012	104	41	63	No	72	91%	74%	34%	76%	61%	26%	54%	Yes (all)	Both	Selective shunting
Felli et al^[^ ^17^ ^]^	2012	300	150	150	No	-	-	-	-	-	-	-	50%	Yes (all)	Both	-
Cho et al^[^ ^15^ ^]^	2014	45	16	29	No	70	77%	84%	27%	16%	38%	-	-	-	-	-
Kuliha et al^[^ ^12^ ^]^	2015	150	77	73	Yes	66	70%	87%	43%	70%	26%	38%	58%	Yes (96%)	Closed cell	Selective shunting

*This number indicates the total number of CAS and CEA procedures performed in each study and for which the DWI is available.

**The reported characteristics refer to the overall population included in the original study and not to the 58 included in the meta-analysis because of DWI availability.

Abbreviations: CAD = coronary artery disease; CAS = carotid artery stenting; CEA = carotid endarterectomy; EPD = embolic protection device; ICSS-MRI = international carotid stenting study-magnetic resonance imaging; RCT = randomized clinical trial

### New DWI lesions

**Fig 2:**
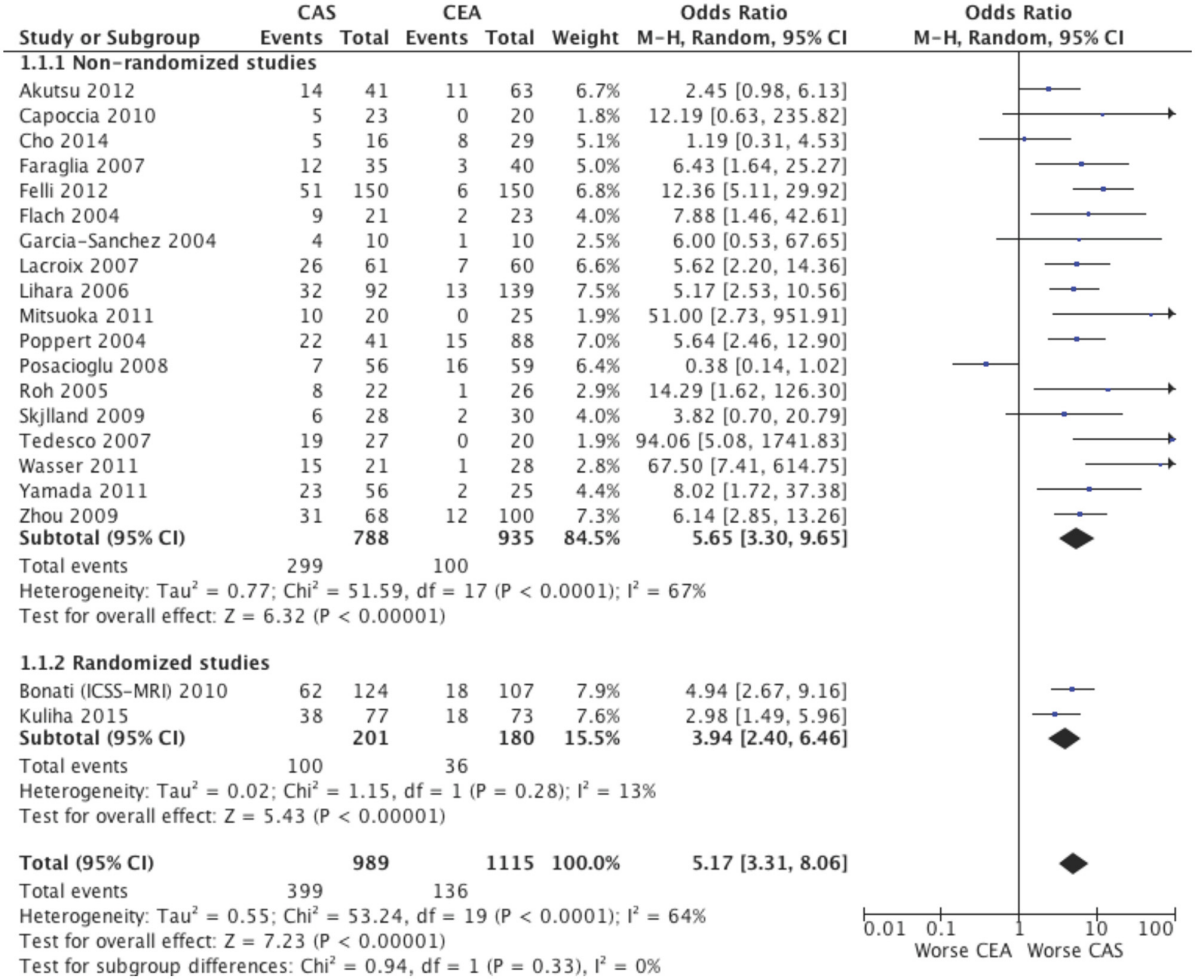
New DWI cerebral lesions after CAS and CEA. Random effects odds ratio and 95% confidence interval for the primary endpoint of new ischemic lesions at DWI after CAS and CEA.

The incidence of new DWI lesions was significantly increased with CAS compared with CEA (40.3% [399 of 989] versus 12.2% [136 of 1115]; 20 studies; 2104 patients; OR 5.17; 95% CI, 3.31–8.06; p<0.00001; Table B in S1 file, Fig 2). The significance was observed both in non-randomized studies (18 studies; 1723 patients; OR 5.65; 95% CI, 3.30–9.65; p<0.00001; Fig 2) and randomized studies (2 studies; 381 patients; OR 3.94; 95% CI, 2.40–6.46; p<0.00001; Fig 2), with a non-significant difference between these subgroups (p for interaction = 0.33; Fig 2).

### Clinical events

Post-procedural death was reported only in two patients of the CAS group and in none of the CEA group (Table B in S1 file).

**Fig 3:**
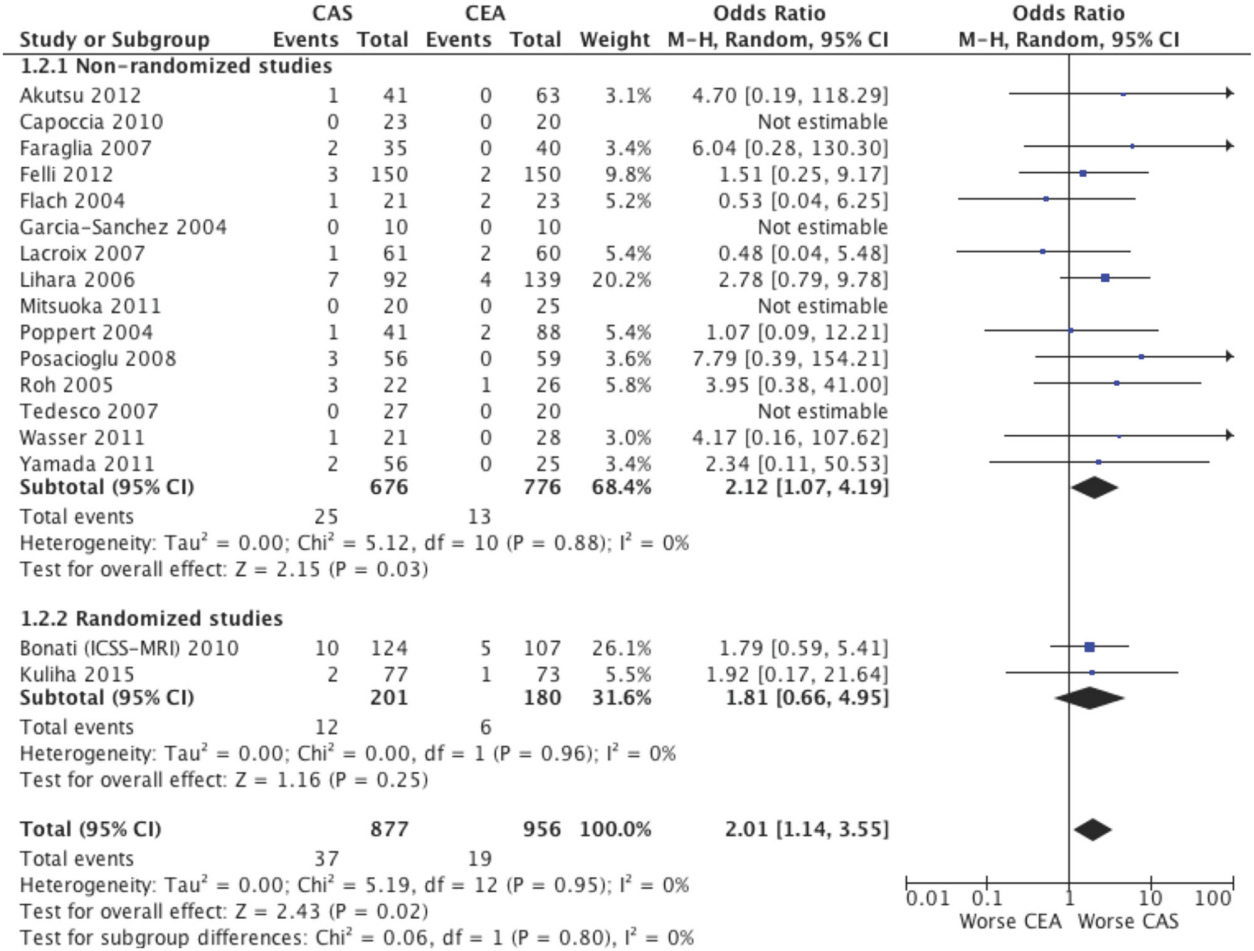
Post-procedural stroke after CAS and CEA. Random effects odds ratio and 95% confidence interval for the post-procedural incidence of stroke after CAS and CEA.

Stroke was significantly higher after CAS then CEA (17 studies; 1833 patients; OR 2.01; 95% CI, 1.14–3.55; p = 0.02; Fig 3). Also stroke or TIA was significantly increased after CAS (17 studies; 1833 patients; OR 2.40; 95% CI, 1.42–4.08; p = 0.001; Fig A in S1 file). In order to include in the analysis those studies in which both CAS and CEA groups did not experience cerebrovascular events, also risk differences were calculated for stroke (RD 0.02; 95% CI, 0.00–0.03; p = 0.04; Fig B in S1 file) and stroke or TIA (RD 0.03; 95% CI, 0.01–0.04; p = 0.002; Fig C in S1 file), however, despite the greater incidence in CAS patients was mitigated, the significances were not altered. The clinical advantage on stroke and stroke or TIA in the CEA group over CAS was also tempered when CEA procedures were performed with shunting in all instead of selective shunting (Figs D and E in S1 file) or when CAS was performed with only closed cell stents instead of both closed cell and open cell stents (Figs F and G in S1 file), however, no significant differences between subgroups emerged (all p for interaction ≥ 0.05).

### Meta-regression

None of the baseline characteristics analyzed (age, male sex, hypertension, diabetes, dyslipidemia, smoke, coronary artery disease, symptoms and year of publication; Table 1) was found to be significant effect modifier (all p values ≥ 0.05) of the three endpoints endpoint (Table C in S1 file).

### Sensitivity Analysis

The overall effect size (OR) of new DWI lesion, stroke and stroke or TIA was confirmed when meta-analyses were repeated removing 1 study at the time (Table D in S1 file).

### Publication bias

The funnel plots and Egger’s test (p = 0.15, p = 0.77 and p = 0.93 for new DWI lesion, stroke and stroke or TIA respectively) did not show any significant publication bias, in all the analyses performed. Trim and fill method confirmed the absence of significant publication bias for new DWI lesion (7 studies trimmed, OR 3.54 2.26–5.56; p<0.0001), stroke (0 studies trimmed, OR 1.9 1.1–3.28, p = 0.02) and stroke or TIA (1 study trimmed, OR 2.22 1.33–3.73, p = 0.001).

## Discussion

The present updated meta-analysis focused on new cerebral ischemic lesions detected by DW-MRI showing that they occur more frequently after CAS than CEA (40.3% versus 12.2%). Moreover, an increased post-procedural rate of ischemic cerebrovascular events (stroke or TIA) was observed in the studies included in this meta-analysis.

A previous meta-analysis including only 6 studies showed that, despite new DWI lesions were significantly increased in CAS versus CEA, the incidence of stroke or death was non-significantly increased [5]. Instead, here we updated incidences of new ischemic events based on the enlarged availability of contemporary data, we confirmed that CAS is associated with more microembolizations, but we also showed increase of stroke or TIA. However, this latter result should be considered with caution because it is restricted to post-procedural time and because it is obtained from clinical events reported only in the studies included in the present meta-analysis, while here we did not include all the events reported in the clinical studies without analysis of new DWI lesions. Moreover, these clinical events were not uniformly reported. Indeed, in some studies stroke was only reported as “all stroke” while in other studies a more detailed sub-classification (major/minor or the disabling/non-disabling) was also included (Table B in S1 file). On the other hand, this result is consistent with the increase of stroke and the long-term superiority of CEA observed in a previous meta-analysis of 13 randomized trials directly comparing CAS and CEA [4].

The results here described should be interpreted on the basis of the following considerations.

First, several studies included in this analysis are dated and the CAS procedures have increasingly ameliorated during these years [3]. In particular, the use of embolic protection devices and closed cell stents instead of open cell stents is currently recommended for CAS. Indeed, in some studies included, EPD were not used, and almost of the studies used distal EPD, therefore the contemporary use of EPD and the diffusion of proximal EPD could reduce the incidence of new lesions and cerebrovascular events [6, 7]. However, in the present meta-analysis, the available data (Table 1) did not allow to compare results according to the use of filter versus proximal occlusion nor to adequately explore the impact of stent type (open cell versus closed cell stents) on CAS results. The risks of CAS have decreased over time due to the improvements in techniques, devices, training and a better selection of patients over time. Accordingly, guidelines recommend that CAS should be performed by centers and operators with a great experience and an adequate number of procedures per year [1]. On the other hand, also for CEA a better outcome could be related to the surgical technique, in particular the selective shunting seems to be better than shunt in all procedures.

Second, it is important to mention that the present study was not designed to generally compare CEA and CAS for all outcomes, therefore all the complications of CEA (such as myocardial infarction, cranial nerve injury, etc) have not been considered.

Third, the impact of these new DWI lesions remains to be defined, indeed, the post-procedural TIA or stroke events are very small compared to the incidence of new DWI lesions, and often also observed in patients without DWI lesions [14, 28]. Moreover, the long-term clinical impact of new cerebral lesions (symptoms, cognitive impairment, etc.) remains debated. Indeed, some data indicate that several of these lesions remain asymptomatic during the follow-up, while according to others show they are associated to clinically relevant consequences.

Fourth, the number, the volume and the localization (ipsilateral, contralateral or bilateral) of new lesions seem to be greater after CAS and seem to have an important role on the long-term outcomes [14–16, 19–22, 24, 26, 28, 30, 32]. Unfortunately, it was not possible to compare these aspects in the present study due to the heterogeneity of few data of direct comparison currently available.

Fifth, it cannot be excluded that differences in the baseline characteristics of patients undergoing CAS or CEA could have affected the results described because the present study comprehensively analyzes the unadjusted incidence of new brain lesions and the majority of studies included are non-randomized. Therefore, whether the increased number of new lesions after CAS was because more patients who had high cardiovascular risk profiles were assigned to stenting is unclear. However, the analysis from the only 2 randomized trials is consistent with the overall result showing a significant increased number of new microembolizations after CAS (OR 3.94; p<0.00001; Fig 2).

Although some data question the equivalence of CAS and CEA, no doubts exist about CAS advantages (minimal invasiveness, absence of general anesthesia and surgical incision, absence of some complications as cranial nerve injury and wound problems, etc.) and its very important value for specific clinical settings (i.e. high risk or inoperable patients, restenosis after CEA, previous neck surgery or radiation therapy, anatomical high bifurcation or extended lesions) [1, 3, 34].

Despite the limitations of the present study, mainly related to differences in study design [35], endpoint definitions and publication bias of the original studies included, on the basis of the results reported, the CAS procedure should be considered at higher risk of procedural ischemic complications compared to CEA. However, the role of these results on the choice of treatment strategy will be increased when the long-term clinical impact of microembolizations will be definitively clarified in larger studies.

## Conclusion

CAS is associated with an increased incidence of new post-procedural DWI lesions compared with CEA (40.3% vs 12.2%; OR 5.17; 95% CI, 3.31–8.06; p<0.00001). This greater amount of ischemic burden may also reflect a higher rate of cerebral events after CAS. However, whether recent technical advances mainly in the field of CAS could potentially reduce these adverse ischemic events still remains to be evaluated.

## Supporting Information

S1 File Supplementary information including: PRISMA Checklist **(Table A)**, post-procedural clinical events reported in the studies included **(Table B)**, meta-regression analyses for the primary endpoint (new cerebral DWI lesions) and secondary endpoints (stroke and stroke or TIA) **(Table C)**, sensitivity analyses for the primary endpoint (new cerebral DWI lesions) and secondary endpoints (stroke and stroke or TIA) **(Table D)**, odds ratio for Stroke of TIA **(Fig. A)**, risk difference for Stroke **(Fig. B)**, risk difference for Stroke of TIA **(Fig. C)**, odds ratio for Stroke according to subgroups of CEA procedure **(Fig. D)**, odds ratio for Stroke or TIA according to subgroups of CEA procedure **(Fig. E)**, odds ratio for Stroke according to subgroups of stent type used in CAS **(Fig. F)**, odds ratio for Stroke or TIA according to subgroups of stent type used in CAS **(Fig. G)** and supplementary references.
(DOCX)
