# Supplementary material for: New Cerebral Lesions at Magnetic Resonance Imaging after Carotid Artery Stenting Versus Endarterectomy: An Updated Meta-Analysis
Source: PLoS One. 2015 May 27;10(5):e0129209. doi: 10.1371/journal.pone.0129209 (PMC4446340; doi:10.1371/journal.pone.0129209)
Supplement: S1 File — Supplementary information including: PRISMA Checklist (Table A), post-procedural clinical events reported in the studies included (Table B), meta-regression analyses for the primary endpoint (new cerebral DWI lesions) and secondary endpoints (stroke and stroke or TIA) (Table C), sensitivity analyses for the primary endpoint (new cerebral DWI lesions) and secondary endpoints (stroke and stroke or TIA) (Table D), odds ratio for Stroke of TIA (Fig. A), risk difference for Stroke (Fig. B), risk difference for Stroke of TIA (Fig. C), odds ratio for Stroke according to subgroups of CEA procedure (Fig. D), odds ratio for Stroke or TIA according to subgroups of CEA procedure (Fig. E), odds ratio for Stroke according to subgroups of stent type used in CAS (Fig. F), odds ratio for Stroke or TIA according to subgroups of stent type used in CAS (Fig. G) and supplementary references. (DOCX) [file pone.0129209.s001.docx]

**NEW CEREBRAL LESIONS AT MAGNETIC RESONANCE IMAGING AFTER CAROTID ARTERY STENTING VERSUS ENDARTERECTOMY: AN UPDATED META-ANALYSIS.**

**SUPPLEMENTARY FILE 1**

**TABLE OF CONTENTS**

**Page 2-3. Table A. PRISMA checklist.**

**Page 4. Table B. Post-procedural clinical events reported in the studies included.**

**Page 5. Table C. Meta-regression analyses for the primary endpoint (new cerebral DWI lesions) and secondary endpoints (stroke and stroke or TIA).**

**Page 6. Table D. Sensitivity analyses for the primary endpoint (new cerebral DWI lesions) and secondary endpoints (stroke and stroke or TIA).**

**Page 7. Fig. A. Odds Ratio for Stroke of TIA.**

**Page 8. Fig. B. Risk Difference for Stroke.**

**Page 9. Fig. C. Risk Difference for Stroke of TIA.**

**Page 10. Fig. D. Odds Ratio for Stroke according to subgroups of CEA procedure.**

**Page 11. Fig. E. Odds Ratio for Stroke or TIA according to subgroups of CEA procedure.**

**Page 12. Fig. F. Odds Ratio for Stroke according to subgroups of stent type used in CAS.**

**Page 13. Fig. G. Odds Ratio for Stroke or TIA according to subgroups of stent type used in CAS.**

**Page 14-16. Supplementary References.**

**Table A. PRISMA checklist.**

| **Section/topic** | **#** | | **Checklist item** | | **Reported on page #** | |  |
| --- | --- | --- | --- | --- | --- | --- | --- |
| **TITLE** | | | | |  | |  |
| Title | 1 | | Identify the report as a systematic review, meta-analysis, or both. | | 1 | |  |
| **ABSTRACT** | | | | |  | |  |
| Structured summary | 2 | | Provide a structured summary including, as applicable: background; objectives; data sources; study eligibility criteria, participants, and interventions; study appraisal and synthesis methods; results; limitations; conclusions and implications of key findings; systematic review registration number. | | 2 | |  |
| **INTRODUCTION** | | | | |  | |  |
| Rationale | 3 | | Describe the rationale for the review in the context of what is already known. | | 3 | |  |
| Objectives | 4 | | Provide an explicit statement of questions being addressed with reference to participants, interventions, comparisons, outcomes, and study design (PICOS). | | 3 | |  |
| **METHODS** | | | | |  | |  |
| Protocol and registration | 5 | | Indicate if a review protocol exists, if and where it can be accessed (e.g., Web address), and, if available, provide registration information including registration number. | | - | |  |
| Eligibility criteria | 6 | | Specify study characteristics (e.g., PICOS, length of follow-up) and report characteristics (e.g., years considered, language, publication status) used as criteria for eligibility, giving rationale. | | 4 | |  |
| Information sources | 7 | | Describe all information sources (e.g., databases with dates of coverage, contact with study authors to identify additional studies) in the search and date last searched. | | 4 | |  |
| Search | 8 | | Present full electronic search strategy for at least one database, including any limits used, such that it could be repeated. | | 4 | |  |
| Study selection | 9 | | State the process for selecting studies (i.e., screening, eligibility, included in systematic review, and, if applicable, included in the meta-analysis). | | 4 | |  |
| Data collection process | 10 | | Describe method of data extraction from reports (e.g., piloted forms, independently, in duplicate) and any processes for obtaining and confirming data from investigators. | | 4 | |  |
| Data items | 11 | | List and define all variables for which data were sought (e.g., PICOS, funding sources) and any assumptions and simplifications made. | | 4 | |  |
| Risk of bias in individual studies | 12 | | Describe methods used for assessing risk of bias of individual studies (including specification of whether this was done at the study or outcome level), and how this information is to be used in any data synthesis. | | - | |  |
| Summary measures | 13 | | State the principal summary measures (e.g., risk ratio, difference in means). | | 4-5 | |  |
| Synthesis of results | 14 | | Describe the methods of handling data and combining results of studies, if done, including measures of consistency (e.g., I^2^) for each meta-analysis. | | 4-5 | |  |
| Section/topic | # | | Checklist item | | Reported on page # | |  |
| Risk of bias across studies | 15 | | Specify any assessment of risk of bias that may affect the cumulative evidence (e.g., publication bias, selective reporting within studies). | | 4-5 | |  |
| Additional analyses | 16 | | Describe methods of additional analyses (e.g., sensitivity or subgroup analyses, meta-regression), if done, indicating which were pre-specified. | | 4-5 | |  |
| **RESULTS** | | | | | |  | |
| Study selection | | 17 | | Give numbers of studies screened, assessed for eligibility, and included in the review, with reasons for exclusions at each stage, ideally with a flow diagram. | | 6-7 | |
| Study characteristics | | 18 | | For each study, present characteristics for which data were extracted (e.g., study size, PICOS, follow-up period) and provide the citations. | | 6-7 | |
| Risk of bias within studies | | 19 | | Present data on risk of bias of each study and, if available, any outcome level assessment (see item 12). | | - | |
| Results of individual studies | | 20 | | For all outcomes considered (benefits or harms), present, for each study: (a) simple summary data for each intervention group (b) effect estimates and confidence intervals, ideally with a forest plot. | | 6-7 | |
| Synthesis of results | | 21 | | Present results of each meta-analysis done, including confidence intervals and measures of consistency. | | 6-7 | |
| Risk of bias across studies | | 22 | | Present results of any assessment of risk of bias across studies (see Item 15). | | 6-7 | |
| Additional analysis | | 23 | | Give results of additional analyses, if done (e.g., sensitivity or subgroup analyses, meta-regression [see Item 16]). | | 6-7 | |
| **DISCUSSION** | | | | | |  | |
| Summary of evidence | | 24 | | Summarize the main findings including the strength of evidence for each main outcome; consider their relevance to key groups (e.g., healthcare providers, users, and policy makers). | | 8 | |
| Limitations | | 25 | | Discuss limitations at study and outcome level (e.g., risk of bias), and at review-level (e.g., incomplete retrieval of identified research, reporting bias). | | 8-10 | |
| Conclusions | | 26 | | Provide a general interpretation of the results in the context of other evidence, and implications for future research. | | 10 | |
| **FUNDING** | | | | | |  | |
| Funding | | 27 | | Describe sources of funding for the systematic review and other support (e.g., supply of data); role of funders for the systematic review. | | 10 | |

**Table B. Outcomes reported in the studies included in the meta-analysis.**

|  | **New DWI lesions** | | **Stroke** | | **Stroke major/disabling** | | **Stroke minor/non-disabling** | | **TIA** | | **Death** | |  |
| --- | --- | --- | --- | --- | --- | --- | --- | --- | --- | --- | --- | --- | --- |
|  | **CAS** | **CEA** | **CAS** | **CEA** | **CAS** | **CEA** | **CAS** | **CEA** | **CAS** | **CEA** | **CAS** | **CEA** | |
| Flach et al ^1^ | 9/21 | 2/23 | 1/21 | 2/23 | 0/21 | 1/23 | 1/21 | 1/23 | 0/21 | 0/23 | 0/21 | 0/23 | |
| Garcia-Sanchez et al ^2^ | 4/10 | 1/10 | 0/10 | 0/10 | 0/10 | 0/10 | 0/10 | 0/10 | 1/10 | 1/10 | 0/10 | 0/10 | |
| Poppert et al ^3^ | 22/41 | 15/88 | 1/41 | 2/88 | - | - | - | - | 0/41 | 0/88 | 0/41 | 0/88 | |
| Roh et al ^4^ | 8/22 | 1/26 | 3/22 | 1/26 | - | - | - | - | 0/22 | 0/26 | 0/22 | 0/26 | |
| Lihara et al ^5^ | 32/92 | 13/139 | 7/92 | 4/139 | 0/92 | 1/139 | 7/92 | 3/139 | 0/92 | 0/139 | 0/92 | 0/139 | |
| Faraglia et al ^6^ | 12/35 | 3/40 | 2/35 | 0/40 | - | - | - | - | 3/35 | 0/40 | 0/35 | 0/40 | |
| Lacroix et al ^7^ | 26/61 | 7/60 | 1/61 | 2/60 | 0/61 | 0/60 | 1/61 | 2/60 | 0/61 | 0/60 | 0/61 | 0/60 | |
| Tedesco et al ^8^ | 19/27 | 0/20 | 0/27 | 0/20 | 0/27 | 0/20 | 0/27 | 0/20 | 3/27 | 0/20 | 0/27 | 0/20 | |
| Posacioglu et al^9^ | 7/56 | 16/59 | 3/56 | 0/59 | - | - | - | - | 0/56 | 0/59 | 1/56 | 0/59 | |
| Skjlland et al ^10^ | 6/28 | 2/30 | - | - | - | - | - | - | - | - | - | - | |
| Zhou et al ^11^ | 31/68 | 12/100 | - | - | - | - | - | - | - | - | 0/68 | 0/100 | |
| Bonati (ICSS-MRI) et al ^12^ | 62/124 | 18/107 | 10/124 | 5/107 | 3/124 | 3/107 | 7/124 | 2/107 | 3/124 | 0/107 | 1/124 | 0/107 | |
| Capoccia et al ^13^ | 5/23 | 0/20 | 0/23 | 0/20 | 0/23 | 0/20 | 0/23 | 0/20 | 0/23 | 0/20 | 0/23 | 0/20 | |
| Mitsuoka et al ^14^ | 10/20 | 0/25 | 0/20 | 0/25 | 0/20 | 0/25 | 0/20 | 0/25 | 1/20 | 0/25 | 0/20 | 0/25 | |
| Wasser et al ^15^ | 15/21 | 1/28 | 1/21 | 0/28 | 0/21 | 0/28 | 1/21 | 0/28 | 0/21 | 0/28 | 0/21 | 0/28 | |
| Yamada et al ^16^ | 23/56 | 2/25 | 2/56 | 0/25 | - | - | - | - | 0/56 | 0/25 | 0/56 | 0/25 | |
| Akutsu et al ^17^ | 14/41 | 11/63 | 1/41 | 0/63 | - | - | - | - | 0/41 | 0/63 | 0/41 | 0/63 | |
| Felli et al ^18^ | 51/150 | 6/150 | 3/150 | 2/150 | 0/150 | 0/150 | 3/150 | 2/150 | 2/150 | 0/150 | 0/150 | 0/150 | |
| Cho et al ^19^ | 5/16 | 8/29 | - | - | - | - | - | - | - | - | - | - | |
| Kuliha et al ^20^ | 38/77 | 18/73 | 2/77 | 1/73 | - | - | - | - | 2/77 | 1/73 | 0/77 | 0/73 | |

Abbreviations: CAS=carotid artery stenting; CEA=carotid endarterectomy; DWI=diffuse-weighted imaging; ICSS-MRI= international carotid stenting study-magnetic resonance imaging; TIA=transient ischemic attack

**Table C. Meta-regression analyses for each outcome.**

|  | **New DWI lesions** | **Stroke** | **Stroke or TIA** |
| --- | --- | --- | --- |
|  | **P value** | **P value** | **P value** |
| Age | 0.93 | 0.73 | 0.98 |
| Male sex | 0.23 | 0.45 | 0.57 |
| Hypertension | 0.84 | 0.65 | 0.56 |
| Diabetes | 0.22 | 0.78 | 0.45 |
| Dyslipidemia | 0.20 | 0.22 | 0.62 |
| Smoke | 0.30 | 0.79 | 0.51 |
| CAD | 0.36 | 0.71 | 0.36 |
| Symptoms | 0.77 | 0.42 | 0.46 |
| Year of publication | 0.60 | 0.78 | 0.51 |

**Table D. Sensitivity analyses for each outcome.**

|  | **New DWI lesions** | | **Stroke** | | **Stroke or TIA** | |
| --- | --- | --- | --- | --- | --- | --- |
|  | **OR** | **P value** | **OR** | **P value** | **OR** | **P value** |
| Flach et al ^1^ | 5.09 | <0.0001 | 2.03 | 0.013 | 2.53 | 0.001 |
| Garcia-Sanchez et al ^2^ | 5.16 | <0.0001 | 1.93 | 0.019 | 2.43 | 0.001 |
| Poppert et al ^3^ | 5.19 | <0.0001 | 1.96 | 0.018 | 2.45 | 0.001 |
| Roh et al ^4^ | 5.02 | <0.0001 | 1.83 | 0.035 | 2.30 | 0.002 |
| Lihara et al ^5^ | 5.25 | <0.0001 | 1.74 | 0.071 | 2.28 | 0.005 |
| Faraglia et al ^6^ | 5.13 | <0.0001 | 1.83 | 0.032 | 2.22 | 0.003 |
| Lacroix et al ^7^ | 5.18 | <0.0001 | 2.05 | 0.012 | 2.55 | 0.001 |
| Tedesco et al ^8^ | 4.86 | <0.0001 | 1.94 | 0.018 | 2.30 | 0.002 |
| Posacioglu et al^9^ | 5.65 | <0.0001 | 1.81 | 0.035 | 2.27 | 0.002 |
| Skjlland et al ^10^ | 5.26 | <0.0001 | - | - | - | - |
| Zhou et al ^11^ | 5.16 | <0.0001 | - | - | - | - |
| Bonati (ICSS-MRI) et al ^12^ | 5.29 | <0.0001 | 1.94 | 0.037 | 2.35 | 0.005 |
| Capoccia et al ^13^ | 5.09 | <0.0001 | 1.93 | 0.019 | 2.40 | 0.001 |
| Mitsuoka et al ^14^ | 4.93 | <0.0001 | 1.92 | 0.020 | 2.33 | 0.002 |
| Wasser et al ^15^ | 4.75 | <0.0001 | 1.86 | 0.027 | 2.33 | 0.002 |
| Yamada et al ^16^ | 5.08 | <0.0001 | 1.86 | 0.027 | 2.36 | 0.002 |
| Akutsu et al ^17^ | 5.47 | <0.0001 | 1.85 | 0.028 | 2.32 | 0.002 |
| Felli et al ^18^ | 4.82 | <0.0001 | 1.95 | 0.022 | 2.34 | 0.003 |
| Cho et al ^19^ | 5.55 | <0.0001 | - | - | - | - |
| Kuliha et al ^20^ | 5.46 | <0.0001 | 1.90 | 0.024 | 2.39 | 0.001 |

Abbreviations: DWI=diffuse-weighted imaging; ICSS-MRI= international carotid stenting study-magnetic resonance imaging; OR=odds ratio; TIA=transient ischemic attack

**Fig. A. Odds Ratio for Stroke of TIA.**

**
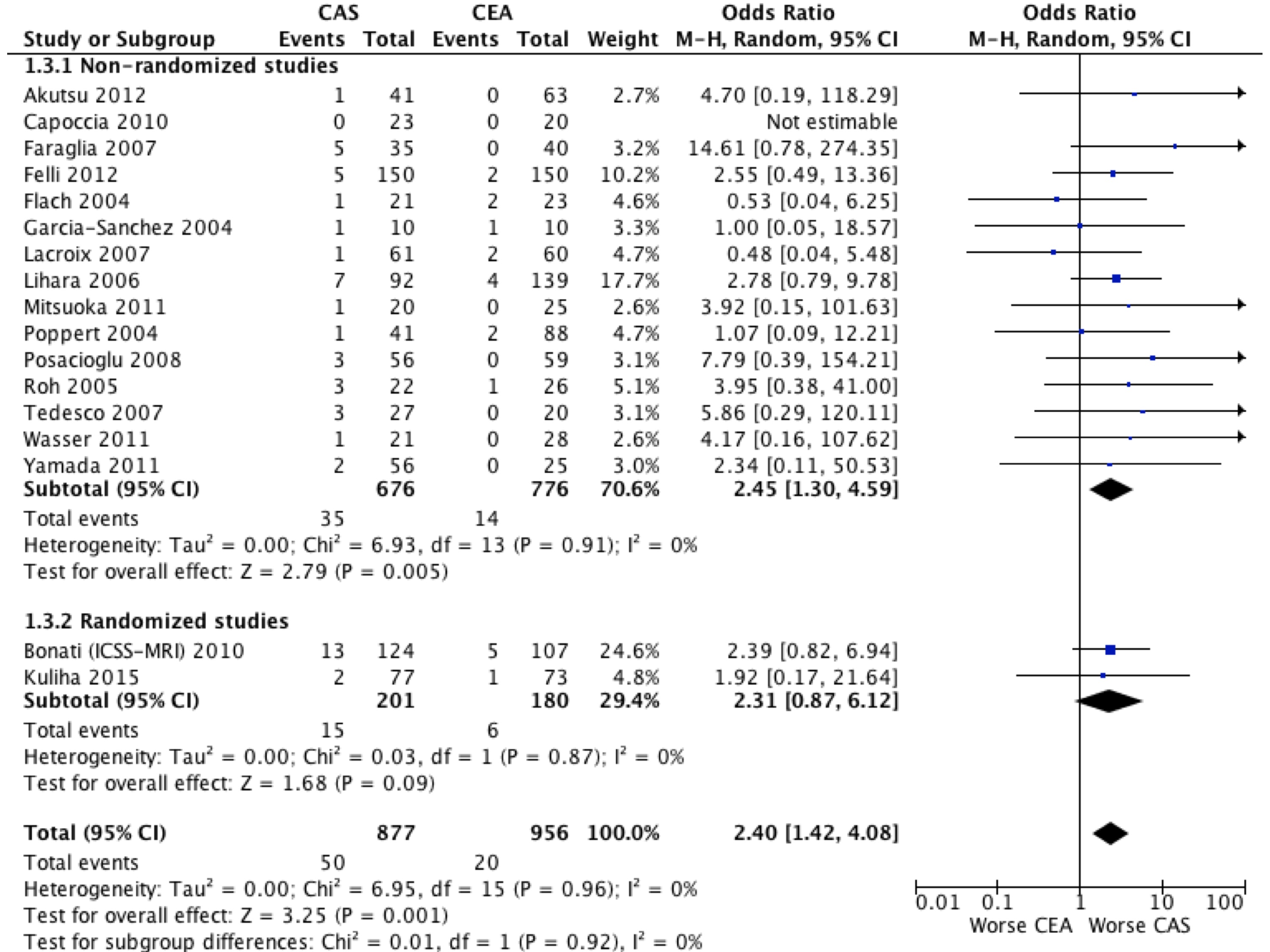
**

**Fig. B. Risk Difference for Stroke.**

**
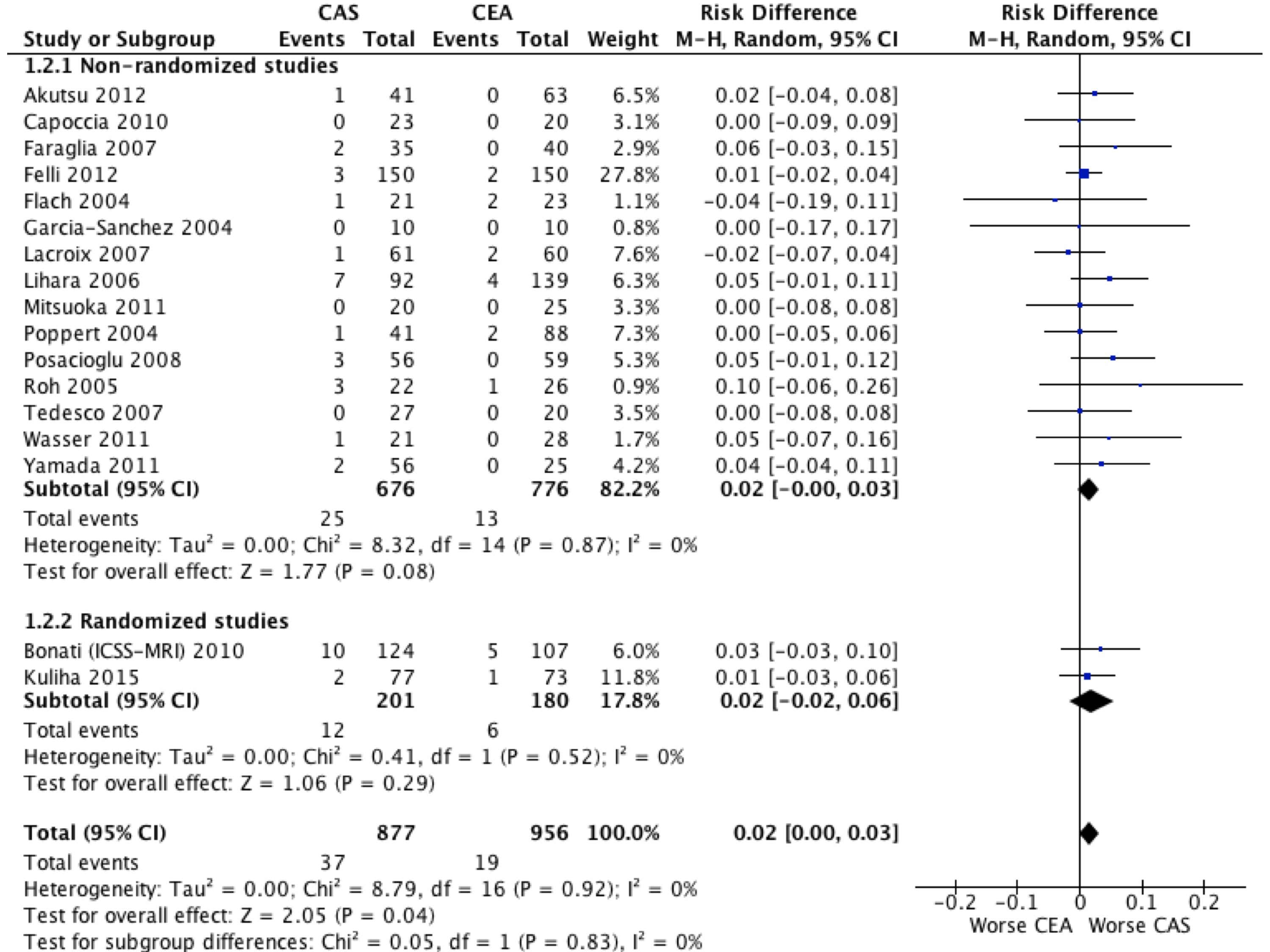
**

**Fig. C. Risk Difference for Stroke of TIA.**

**
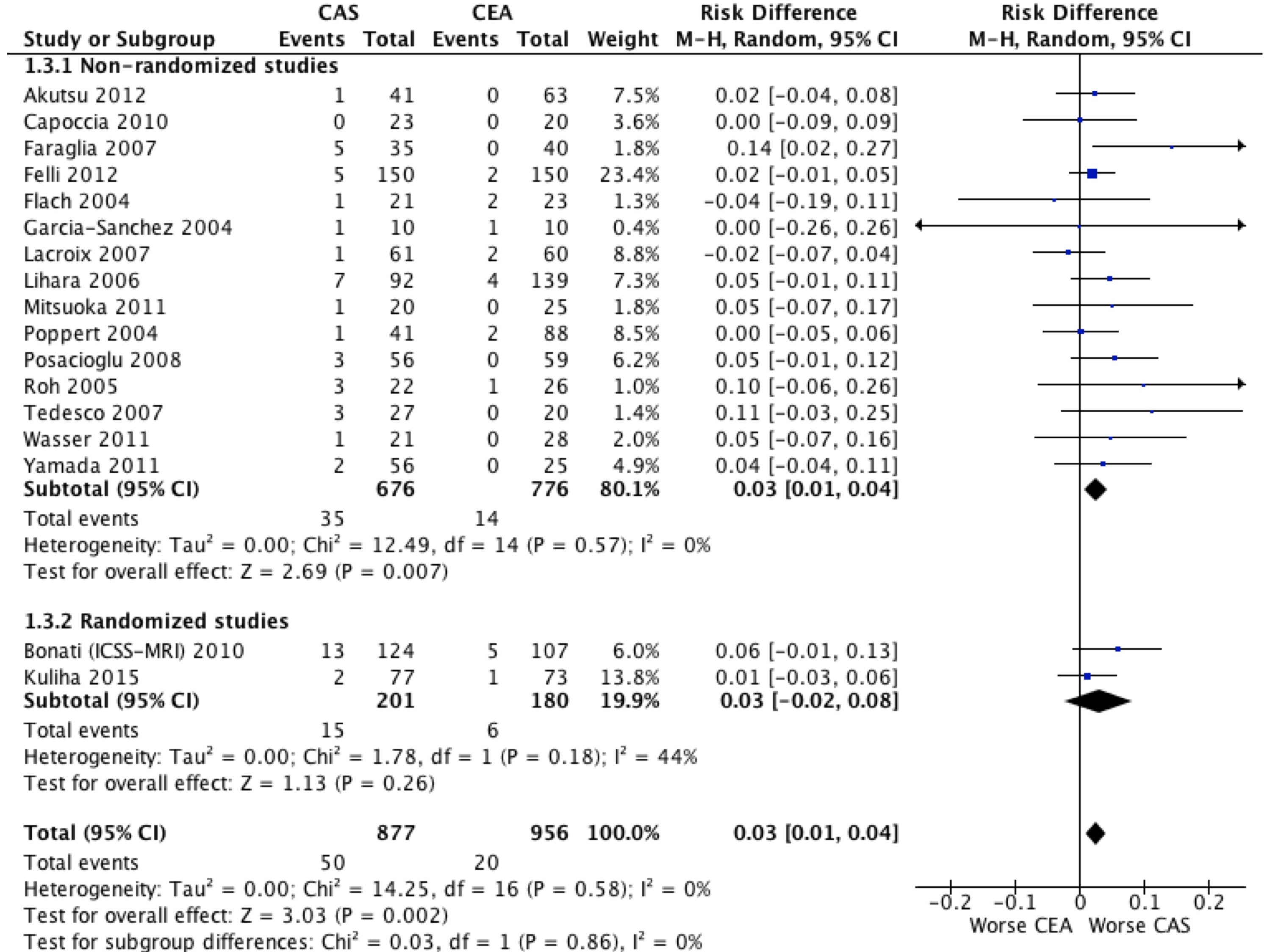
**

**Fig. D. Odds Ratio for Stroke according to subgroups of CEA procedure.**

**
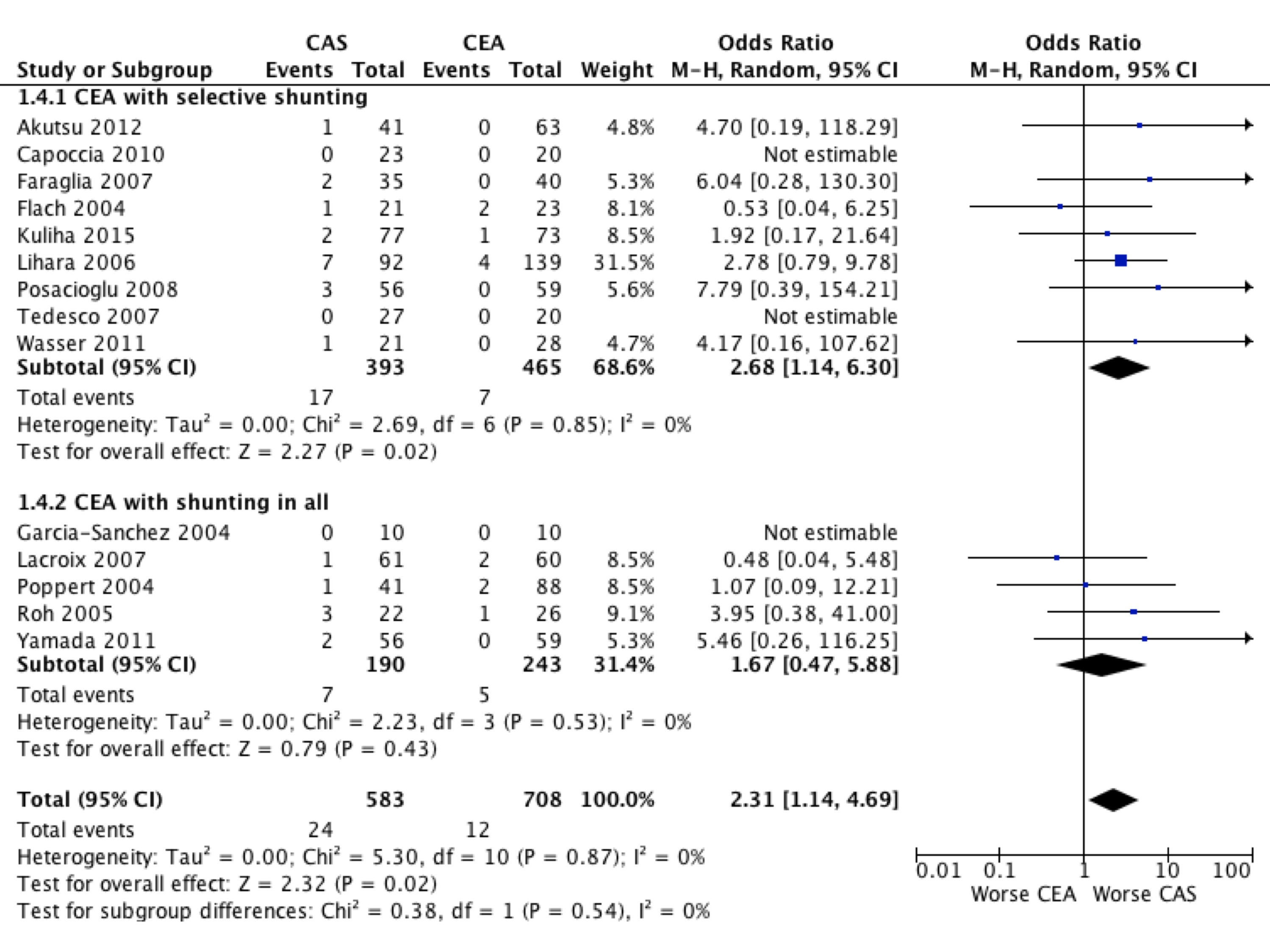
**

**Fig. E. Odds Ratio for Stroke or TIA according to subgroups of CEA procedure.**

**
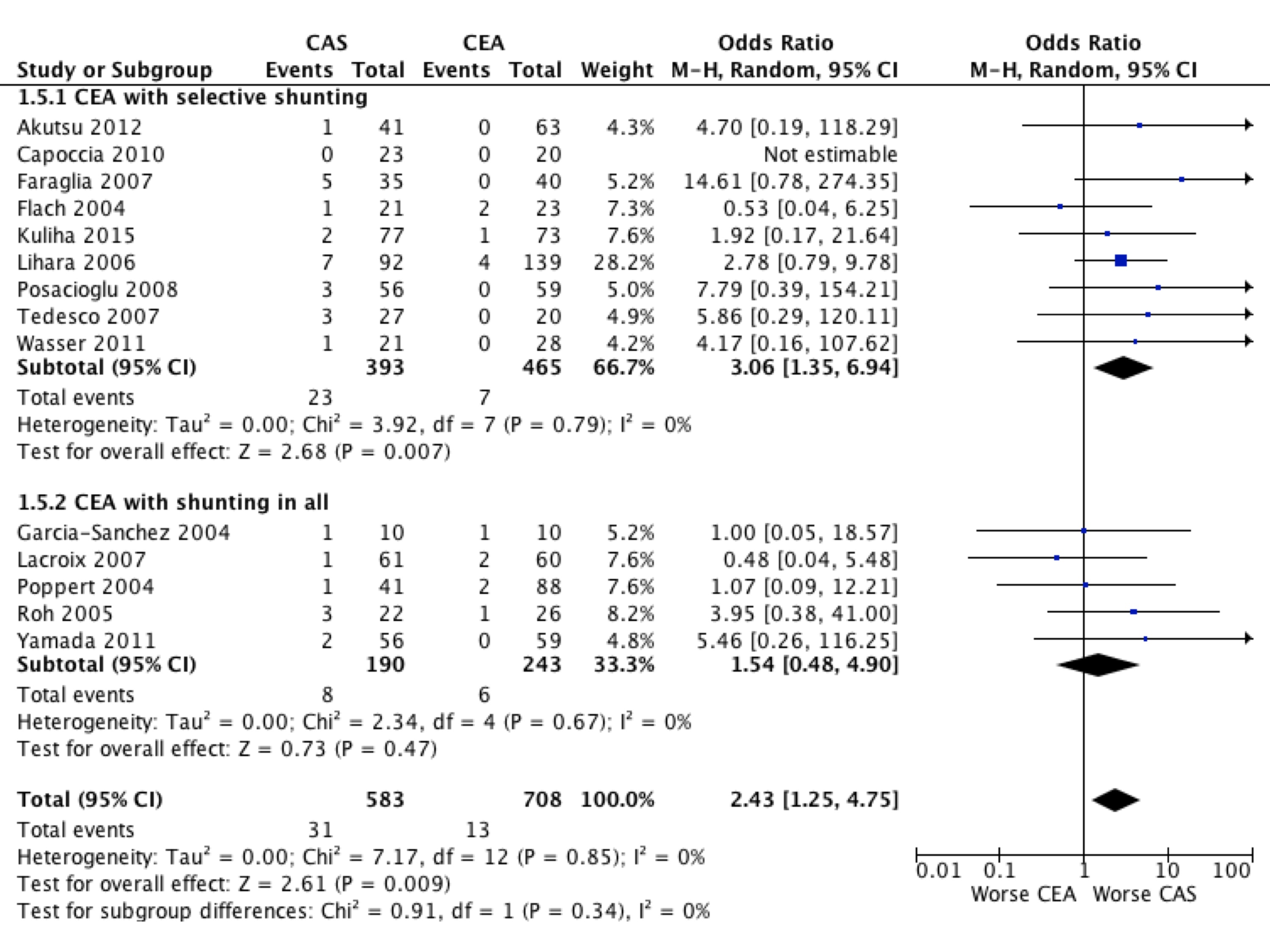
**

**Fig. F. Odds Ratio for Stroke according to subgroups of stent type used in CAS.**

**
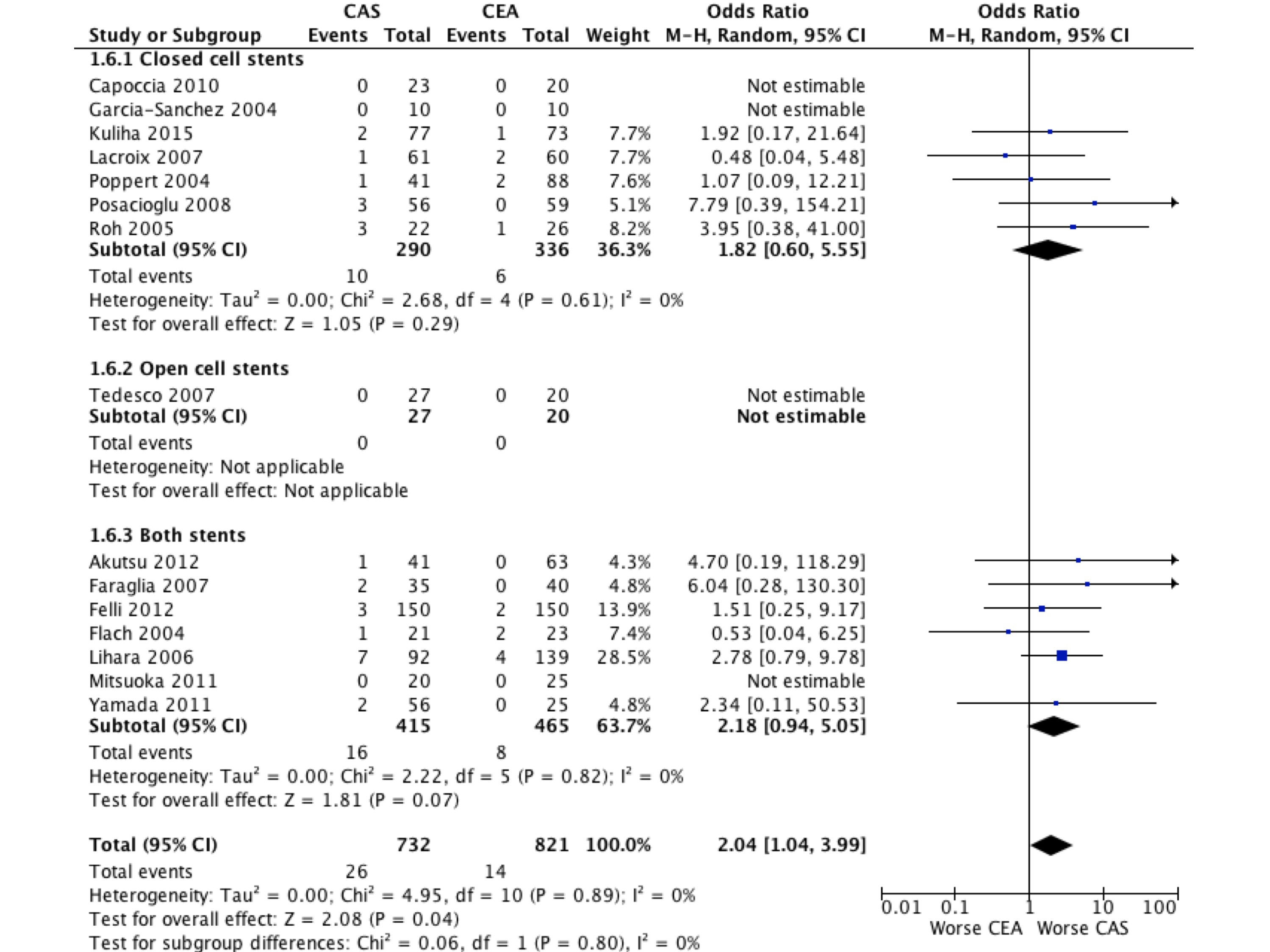
**

**Fig. G. Odds Ratio for Stroke or TIA according to subgroups of stent type used in CAS.**


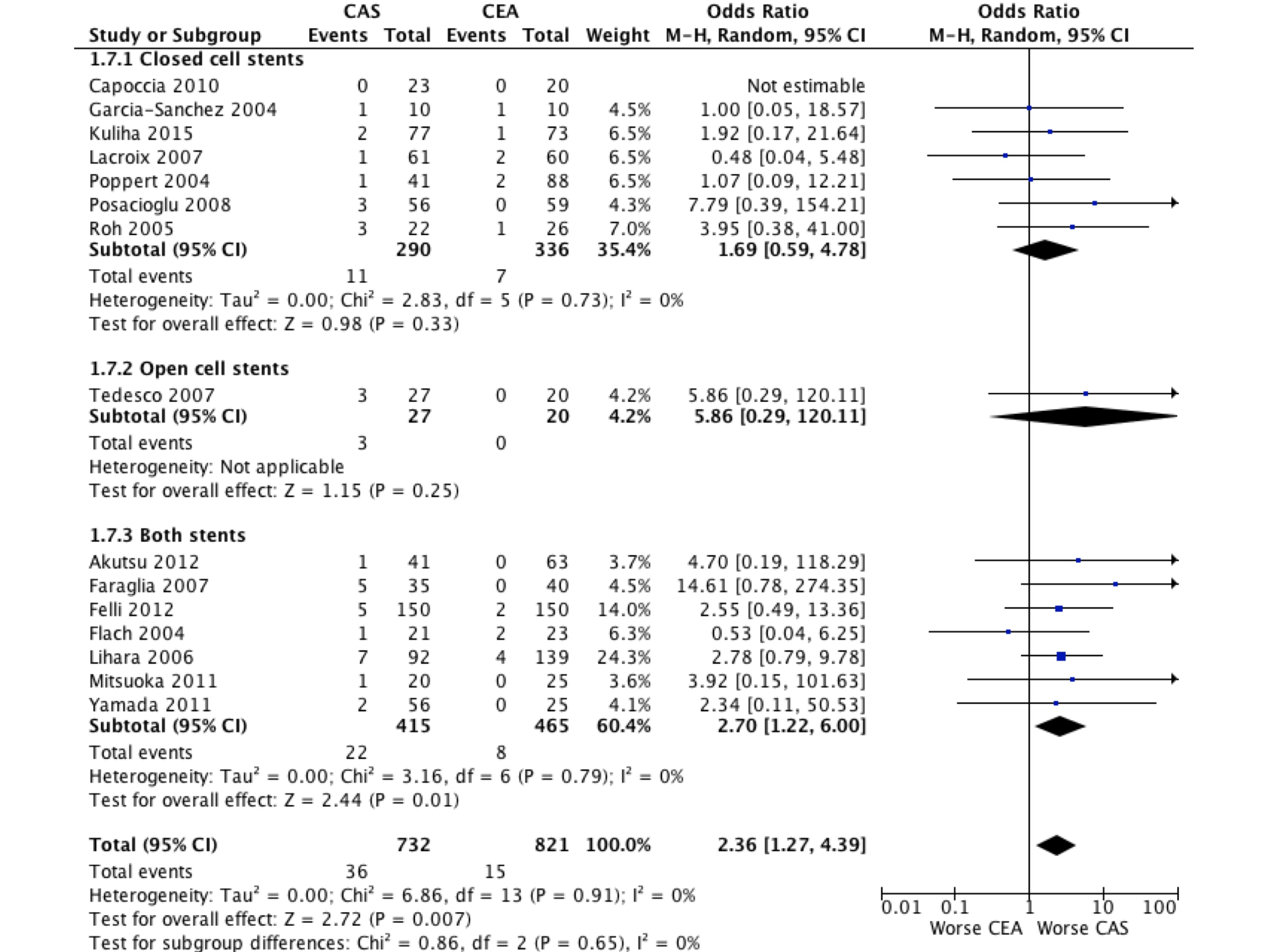


**Supplementary References**

1. Flach HZ, Ouhlous M, Hendriks JM, Van Sambeek MR, Veenland JF, Koudstaal PJ, et al. Cerebral ischemia after carotid intervention. J Endovasc Ther. 2004;11:251-257
2. Garcia-Sanchez S, Millan-Torne M, Capellades-Font J, Muchart J, Callejas PJ, Vila-Moriente N. Ischemic brain lesions following carotid revascularisation procedures: A comparative study using diffusion-weighted magnetic resonance imaging. Rev Neurol. 2004;38:1013-1017
3. Poppert H, Wolf O, Resch M, Theiss W, Schmidt-Thieme T, Graefin von Einsiedel H, et al. Differences in number, size and location of intracranial microembolic lesions after surgical versus endovascular treatment without protection device of carotid artery stenosis. J Neurol. 2004;251:1198-1203
4. Roh HG, Byun HS, Ryoo JW, Na DG, Moon WJ, Lee BB, et al. Prospective analysis of cerebral infarction after carotid endarterectomy and carotid artery stent placement by using diffusion-weighted imaging. AJNR. Am J Neuroradiol. 2005;26:376-384
5. Iihara K, Murao K, Sakai N, Yamada N, Nagata I, Miyamoto S. Outcome of carotid endarterectomy and stent insertion based on grading of carotid endarterectomy risk: A 7-year prospective study. J Neurosurg. 2006;105:546-554
6. Faraglia V, Palombo G, Stella N, Taurino M, Iocca ML, Romano A, et al. Cerebral embolization in patients undergoing protected carotid-artery stenting and carotid surgery. J Cardiovasc Surg. 2007;48:683-688
7. Lacroix V, Hammer F, Astarci P, Duprez T, Grandin C, Cosnard G, et al. Ischemic cerebral lesions after carotid surgery and carotid stenting. Eur J Vasc Surg. 2007;33:430-435
8. Tedesco MM, Lee JT, Dalman RL, Lane B, Loh C, Haukoos JS, et al. Postprocedural microembolic events following carotid surgery and carotid angioplasty and stenting. J Vasc Surg. 2007;46:244-250
9. Posacioglu H, Engin C, Cinar C, Apaydin AZ, Oran I, Parildar M, et al. Carotid endarterectomy versus carotid artery stenting: Findings in regard to neuroclinical outcomes and diffusion-weighted imaging. Tex Heart Inst J. 2008;35:395-401
10. Skjelland M, Krohg-Sorensen K, Tennoe B, Bakke SJ, Brucher R, Russell D. Cerebral microemboli and brain injury during carotid artery endarterectomy and stenting. Stroke. 2009;40:230-234
11. Zhou W, Dinishak D, Lane B, Hernandez-Boussard T, Bech F, Rosen A. Long-term radiographic outcomes of microemboli following carotid interventions. J Vasc Surg. 2009;50:1314-1319
12. Bonati LH, Jongen LM, Haller S, Flach HZ, Dobson J, Nederkoorn PJ, et al. New ischaemic brain lesions on mri after stenting or endarterectomy for symptomatic carotid stenosis: A substudy of the international carotid stenting study (icss). Lancet Neurol. 2010;9:353-362
13. Capoccia L, Speziale F, Gazzetti M, Mariani P, Rizzo A, Mansour W, et al. Comparative study on carotid revascularization (endarterectomy vs stenting) using markers of cellular brain injury, neuropsychometric tests, and diffusion-weighted magnetic resonance imaging. J Vasc Surg. 2010;51:584-591, 591 e581-583; discussion 592
14. Mitsuoka H, Shintani T, Furuya H, Nakao Y, Higashi S. Ultrasonographic character of carotid plaque and postprocedural brain embolisms in carotid artery stenting and carotid endarterectomy. Ann Vasc Dis. 2011;4:106-109
15. Wasser K, Pilgram-Pastor SM, Schnaudigel S, Stojanovic T, Schmidt H, Knauf J, et al. New brain lesions after carotid revascularization are not associated with cognitive performance. J Vas Surg. 2011;53:61-70
16. Yamada K, Yoshimura S, Kawasaki M, Enomoto Y, Asano T, Hara A, et al. Embolic complications after carotid artery stenting or carotid endarterectomy are associated with tissue characteristics of carotid plaques evaluated by magnetic resonance imaging. Atherosclerosis. 2011;215:399-404
17. Akutsu N, Hosoda K, Fujita A, Kohmura E. A preliminary prediction model with mr plaque imaging to estimate risk for new ischemic brain lesions on diffusion-weighted imaging after endarterectomy or stenting in patients with carotid stenosis. AJNR. Am J Neuroradiol. 2012;33:1557-1564
18. Felli MM, Alunno A, Castiglione A, Malaj A, Faccenna F, Jabbour J, et al. Cea versus cas: Short-term and mid-term results. Int Angiol. 2012;31:420-426
19. Cho AH, Cho YP, Lee DH, Kwon TW, Kwon SU, Suh DC, et al. Reperfusion injury on magnetic resonance imaging after carotid revascularization. Stroke. 2014;45:602-604
20. Kuliha M, Roubec M, Prochazka V, Jonszta T, Hrbac T, Havelka J, et al. Randomized clinical trial comparing neurological outcomes after carotid endarterectomy or stenting. Br J Surg. 2015;102:194-201
